# Supervisor support and virtual leadership moderate the association between technostress creators and strain in remote work: Evidence based on hair cortisol and occupational physician’s hetero-evaluations

**DOI:** 10.1371/journal.pone.0323385

**Published:** 2025-06-13

**Authors:** Damiano Girardi, Sebastiano Rapisarda, Elvira Arcucci, Laura Dal Corso, René Riedl, Isabella Pividori, Alessandra Falco

**Affiliations:** 1 Department of Philosophy, Sociology, Education and Applied Psychology, University of Padua, Padua, Italy; 2 Digital Business Institute, University of Applied Sciences Upper Austria, Campus Steyr, Steyr, Austria; 3 Institute of Business Informatics—Information Engineering, University of Linz, Linz, Austria; 4 Department of Agricultural, Environmental and Animal Sciences, University of Udine, Udine, Italy; Universitat de Girona, SPAIN

## Abstract

We investigated the association between technostress creators (TCs) and the strain response among smart workers, who are defined as individuals who make extensive use of information and communication technologies for work-related tasks and in interpersonal relationships with colleagues and supervisors. The moderating role of supervisor support and virtual leadership was a specific focus of our study. We conducted two studies, in each of which we complemented the self-report measures on the TCs and supervisor support and virtual leadership with more objective measures of stress and strain. In Study 1, we investigated the association between perceived TCs and hair cortisol concentration (HCC) as a biomarker of stress. In Study 2, we examined the association between perceived TCs and stress-related psychophysical symptoms (i.e., psychophysical strain) as assessed by the occupational physician (i.e., hetero-evaluation). In Study 1, 102 smart workers from different organizations completed a self-report questionnaire and we collected a strand of hair. Study 1 provided little support for the association between TCs and HCC, but supervisor support did affect the association between techno-uncertainty (one of five TCs) and HCC, which was positive when support was low, but negative—albeit marginally significant—when support was high. In Study 2, 105 smart workers from a company in the service sector completed a self-report questionnaire and underwent an occupational health examination. Techno-invasion (another TC) was positively related to psychophysical strain as assessed by the occupational physician. In addition, virtual leadership attenuated the association between three TCs (techno-overload, techno-invasion, techno-insecurity) and psychophysical strain. Based on these nuanced pattern of results, our studies suggest that TCs lead to a strain response among smart workers, and that positive relationships with one’s supervisor—in terms of supervisor support and virtual leadership—can attenuate the negative consequences of technostress.

## Introduction

In recent decades, information and communication technologies (ICTs) have created new ways of organizing work by providing employers and employees with greater flexibility, in terms of when and where work can be done. This has facilitated the spread of alternative work arrangements—where work that could be done at the employer’s premises is carried out off-site using ICTs [1]—a process that accelerated significantly during the COVID-19 pandemic [2]. Although alternative work arrangements vary in terms of the intensity of time spent working remotely, two key aspects are the physical separation from company premises and the intensive use of ICTs [1]. A common form of alternative work arrangements is smart working (SW), characterized by a high working time and space flexibility and the intensive use of ICTs to carry out tasks and communicate with supervisors/colleagues [3]. Smart working has evolved into a crucial concept in the business world, particularly in the human resources sector. Market research data indicates that the opportunity for SW has become a significant factor in the choice of employers, especially for younger employees [4]. In Italy, SW has even been integrated into law, based on the following definition: SW is “a mode of implementing the employment relationship established by agreement between the parties, also with forms of organization by phases, cycles and objectives and without precise time or place constraints, with the possible use of technological tools for carrying out the work activity” (Law 81/2017). What follows is that current developments signify the importance of SW, and this significance is predicted to further grow in the future [4].

While ICTs can enable greater autonomy in performing one’s work tasks both within and outside the office, the massive use of digital technologies when working remotely may be associated with work intensification, in terms of work overload (e.g., working for longer hours and on irregular schedules), information overload (e.g., large amounts of information from different sources) and the expectation of being always available for work even during leisure time [5]. Not surprisingly, past research has shown an association between ICTs use and stress, psychophysical symptoms, and presenteeism (including virtual presenteeism in digitalized environments), with regular home-based remote working being associated with the highest number of health symptoms [1].

Against this background, in this study we examined the relationship between technology-related risk factors—in the following referred to as technostress creators (TCs)—and the strain response among smart workers (SWs) during the COVID-19 pandemic, with a focus on the protective role of supportive relationships with one’s supervisors. This focus is supported by conceptual arguments in the academic literature on the significance of supportive leadership, which, however, have not been empirically examined so far (e.g., Iannotta et al. [6]). Theoretically, we build on the transactional model of stress [7] and the job demands-resources (JD-R) theory [8] to investigate the associations between TCs and the strain response, in terms of hair cortisol concentration (HCC)—a biomarker of stress [9]—and psychophysical strain, namely psychological and physical symptoms associated with work-related stress [10]. In addition to supervisors’ support (SS) [11], we also studied virtual leadership (VL) [12], conceptualized as a process of social influence mediated by ICTs [13,14]. Virtual leadership is a major determinant of organizational success in an increasingly digital world, as it affects employees’ skills, attitudes, and behaviors in virtual working environments [12]. Virtual leadership and supervisor support are two closely related but distinct concepts. While both refer to the process by which leaders influence and manage their team members, the former pertains specifically to the ability of leaders to manage, inspire, and guide remote teams using digital tools and strategies, with a focus on effective virtual communication. Conversely, supervisor support is more operational in nature and refers to the direct support and guidance that supervisors provide to their employees. We will clarify the concept of VL further in the following sections of the manuscript. Similar to supportive leadership, we are not aware of any empirical research that has investigated VL’s role in the context of SW. However, this lack of empirical research represents a significant research deficit, as both aspects of the positive relationship with one’s supervisor can buffer the association between TCs and the strain response. Importantly, we focused on SWs because they (i) make extensive use of ICTs to perform work-related tasks, (ii) communicate and have virtual interpersonal relationships by means of digital tools, and (iii) likely experience a wide range of technology-related stressful situations at work (e.g., technologies invading private life and spaces in addition to work overload).

### Theoretical background

In recent years, researchers have devoted considerable attention to the phenomenon of technostress [15]. Initially, Brod [16] conceived technostress as a modern disease of adaptation resulting from an inability to effectively handle new information technologies. Later, Ragu-Nathan et al. [17] described technostress as the stress individuals experience because of the use of information systems. According to recent literature [18,19], technostress is a constantly evolving phenomenon—as new technologies are constantly adopted in organizations—as well as an interdisciplinary one, particularly as it involves a theoretical link between the literature on information systems and psychophysiological stress [15]. First, the coming of the COVID-19 pandemic has forced the necessity for employers and employees to adopt remote working (of which SW is a major form), which produced a considerable acceleration in the pervasiveness and use of new digital technologies. Second, given the multifaceted nature of stress—which encompasses a variety of psychological, physiological, and behavioral processes—technostress research benefit from the contributions of different research disciplines [20] and a multi-method approach [21].

From a theoretical standpoint, although several theoretical frameworks have been used in technostress research, the transactional model of stress [7] is frequently adopted in the field, particularly within organizational contexts. In this perspective, stress is conceptualized as a process involving a transaction between the individual and the environment. Specifically, stress encompasses the presence of environmental conditions appraised by individuals as stressors or demands that deplete resources and elicit coping responses which, in turn, give rise of psychological, physiological, and behavioral outcomes [7]. In this perspective, the phenomenon of technostress can be conceived as a process encompassing the existence of technological environmental conditions (i.e., technologies and their characteristics that individuals use throughout the day) that are first evaluated to establish whether this stimulus is of significance. If the conditions are perceived as potentially threatening demands or techno–stressors, coping responses result, either emotion or problem-focused. Lazarus and Folkman [7] describe problem-focused and emotion-focused coping. The former aims to modify the person-environment realities related to a stressful situation, while the latter focuses on reducing negative emotions by altering the appraisal of the stressful situation. An illustration of a problem-focused strategy is enhancing computer knowledge to increase control over potential ICT malfunctions. On the other hand, an example of an emotion-focused strategy involves minimizing the *perceived* negative impact of an ICT problem on achieving a specific task in an organizational context by cognitive reinterpretation [22]. Coping responses may subsequently lead to psychological, physiological, and behavioral outcomes for the individual over time (e.g., exhaustion, increased stress hormone excretion, or altered performance) [15,20].

Past research has focused on different aspects of the technostress process, including the association between threatening TCs and adverse consequences for the individual and the organization [15]. Specifically, the former include techno-overload, when ICTs force workers to work more and faster (“too much”); techno-invasion, when ICTs invades private spaces creating pressures of constant connectivity (“always connected”); techno-complexity, or the complexity associated to ICTs that leads to a sense of inadequacy with regard to computer skills (“too difficult”); techno-insecurity, implying that workers feel threatened about losing their jobs because of ICTs (“being replaced”); and techno-uncertainty, referring to constant technological changes that may create uncertainty and consequently stress for workers (“too many changes”) [19].

According to the JD-R theory, which offers a unifying theoretical framework that integrates work-related stress and motivational perspectives [8], TCs can be conceptualized as job demands (i.e., risk factors), that is, work-related characteristics that require efforts and are associated with psychological and/or physiological costs for the individual [8]. Not surprisingly, TCs may result in outcomes, both at individual and organizational level, which include negative psychological, physiological, and behavioral responses [19,20,23]. Psychological outcomes include cognitive and affective responses such as job burnout (e.g., exhaustion), role conflict, role overload, and reduced job satisfaction [17,19], behavioral outcomes encompass, among others, lower job performance and reduced productivity at organizational level [19,23,24], and physiological responses may include the secretion of stress hormones (e.g., cortisol) and other important precursors (e.g., alpha-amylase, adrenocorticotropic hormone), as well as the increased activation of the sympathetic nervous system, including heart rate and blood pressure [25]. Importantly, sustained or chronic activation of biological stress mechanisms can lead to adverse health effects. The following consequences, among others, are reported in the scientific literature: depression, burnout, insomnia, migraine headaches, bronchial asthma, abdominal obesity, chronic hypertension, coronary heart disease, suppressed immune function, and cancer [10,26–29].

Beyond factors that create stress due to the use of ICTs, there are conditions that have the potential to reduce the level of technostress experienced by ICTs users, such as organizational and technical support, as well as positive, supportive relationships with one’s supervisors [17]. Specifically, SS seems to be helpful in limiting the negative consequences of TCs by helping workers to accomplish task effectively as well as showing that managers have reasonable expectations of employees (e.g., regarding their availability outside of working hours) [30]. Based on the JD-R theory, these factors can be conceived as job resources (i.e., protective factors) since they can reduce the psychophysiological costs associated with techno-stressors. In addition, positive leadership styles were found to mitigate the effect of TCs on job strain [31]. Notably, a recent review suggests that leadership may play different roles in the JD-R theory [32], which include the reduction of negative effects of job demands. For example, a leader may help employees to deal with job demands (e.g., elevated techno-overload) by providing them with valuables job resources (e.g., greater technology-related autonomy) [33]. Hence, based on the above reasoning and the mentioned paucity of corresponding empirical research, in the present study we focused on two specific aspects of the positive relationships with one’s supervisor, namely SS and VL, as factors that can buffer the association between TCs and negative outcomes.

As pointed out by Nastjuk et al. [19], a limitation of past research concerns the measurement of technostress. Specifically, the multi-faceted nature of technostress, which includes a variety of psychological, physiological, and behavioral aspects [15], should be explored through a multi-method approach that combines different measurement methods (e.g., self-/other-report, physiological data, objective data on outcome variables). Despite the articulated significance of a multi-method approach in technostress research [21], corresponding studies are still relatively uncommon in the field (although notable exceptions exist; for a review see Fischer and Riedl [20]). In fact, past research primarily relied on self-report data, whereas physiological data and objective data on outcome variables (e.g., objective performance measures) have been less studied [19]. Specifically, the review by Fischer and Riedl revealed that about one third of technostress research adopted a multi-method approach. Specifically, among the studies that include self-report, 23% also used biological measures, while 7% combined self-report and interviews [20].

### Purpose of the present study

In order to overcome the weakness of a single-method approach (e.g., common method bias) [34], as well as to contribute to a better understanding of the consequences of technostress, we conducted two studies that integrated different measurement methods. In Study 1, we used self-report measures (i.e., survey) to determine TCs in combination with a biomarker of stress, namely HCC [9]. In Study 2, we combined self-report measures (i.e., survey) of TCs with the assessment of psychophysical symptoms carried out by the occupational physician during the occupational health surveillance. These studies together provide a comprehensive understanding of the health effects of TCs, as well a deeper understating on the protective role of positive relationships with one’s supervisor, in terms of SS and VL.

## Study 1: Hair cortisol and the allostatic load model

To better understand the psychophysiological mechanisms underlying the relationship between chronic/prolonged TCs and workers’ physical and mental health, this study builds on the allostatic load model [35], which has also been integrated with the JD-R theory [36]. According to this model, exposure to workplace stressors, including the TCs, is associated with a state of physiological activation that results in the release of primary mediators of the stress response, including stress hormones such as cortisol. When employees are exposed to chronic or prolonged stressful situations (e.g., SWs facing prolonged TCs) without adequate recovery, the sustained activation of primary mediators may occur [37]. Over time, this activation can lead to secondary mediators (e.g., increased blood pressure), ultimately resulting in allostatic overload and psychological or physical outcomes, such as depression or cardiovascular disease [10]. In line with the allostatic load model, TCs—in terms of techno-overload, techno-invasion, techno-complexity, techno-insecurity, and techno-uncertainty—require efforts and are therefore associated with psychophysiological costs for the individual [25]. Accordingly, we hypothesized TCs to be positively associated with the cumulative concentrations of cortisol over time. Specifically, in this study we looked at the cumulative HCC over the previous three months in a sample of SWs. This hypothesis is supported by empirical evidence. Organizational changes caused by the implementation of new ICTs systems may result in significant increases in users’ cortisol concentrations, as assessed via urine samples [38]. Additionally, the perception of a computer breakdown during the execution of a computer-based task may lead to significant increases in users’ cortisol concentrations, as assessed via saliva samples [39].

Hypothesis 1 (H1): Technostress creators, in terms of techno-overload, techno-invasion, techno-complexity, techno-insecurity, and techno-uncertainty, will be positively associated with hair cortisol concentrations.

### The role of supervisor support

As mentioned above, SS—as a job resource—can reduce the costs associated with TCs, which is consistent with the buffer hypothesis of the JD-R theory [8]. For example, supervisors can provide employees working remotely with instrumental resources useful to accomplish technology-related tasks more effectively (e.g., the provision of resources such as feedback and advice may promote a better fit between work tasks and technology) [40]. Similarly, supervisors may help SWs to successfully overcome temporary problems related to the use of ICTs, or they may formulate clear expectations regarding availabilities, which may result in clearer boundaries between work and private life, and therefore in less uncertainty and hence stress [18]. Also, supervisors may provide opportunities for recovery (e.g., permission to participate in stress reduction programs such as yoga during working hours; note that the effectiveness of such programs has been demonstrated empirically for employees who suffer from technostress) [41]. Overall, based on this reasoning, and in line with the buffer hypothesis of the JD-R theory and previous behavioral research [42], we hypothesized that SS helps to reduce the physiological costs associated with the prolonged or chronic exposure to TCs among SWs, thus mitigating the association between TCs and HCC.

Hypothesis 2 (H2): Supervisor support will moderate the association between technostress creators, in terms of techno-overload, techno-invasion, techno-complexity, techno-insecurity, and techno-uncertainty, and hair cortisol concentrations, which is expected to be weaker when supervisor support is high.

### Materials and methods

#### Procedure and participants.

The study was conducted during the COVID-19 pandemic and involved SWs from different organizations in Italy. The recruitment period started on 1 April 2022 and ended on 5 May 2022. A total of 115 SWs were recruited using a snowball sampling technique and invited to take part in a study about wellbeing at work and biomarkers of stress. Overall, 102 SWs agreed to participate. Before proceeding, they were informed that their participation was voluntary and confidential, and that they were free to withdraw at any time. Upon acceptance, they were provided with a link to the online informed consent form, so that all participants provided written informed consent before data collection. Next, SWs completed an online survey (i.e., psychological data) aimed at determining TCs, supervisor support, as well as several sociodemographic variables (sex, age, occupation, seniority, and type of contract). Additionally, workers entered a personal alphanumeric code at the top of the questionnaire, which was necessary to match psychological and biological data. Finally, participants were given detailed instructions on how to collect the biological sample, which was a strand of hair measuring at least 3 cm, taken non-invasively from the vertex of the back of the head (i.e., biological data). The study was performed in line with the principles of the Declaration of Helsinki. The project was approved by the Psychological Research Ethics Committee (Area 17), Department/Section of Psychology – University of Padua, Italy (protocol n. 4635).

The sample consisted of 72 female (70.6%) and 30 male (29.4%) with a mean age of 39.3 years (*SD* = 12.2). With respect to their occupation, 61 participants (59.8%) were white-collar, 26 (25.5%) were managers or self-employed, and 10 (9.8%) were schools professionals. In terms of seniority, 52 (51%) participants had been with their current company for less than 5 years, while 22 (21.6%) had been with their current company for 20 years or more. Finally, 73 participants (71.6%) had a permanent contract, while 29 had a fixed term one (28.4%).

#### Measures.

With respect to psychological data, the self-report questionnaire included the following measures:

Technostress creators were determined using an Italian adaptation of the questionnaire developed by Ragu-Nathan et al. [17]. The scale included 20 items that reflect techno-overload (four items, e.g., I am forced by this technology to do more work than I can handle), techno-invasion (three items, e.g., I feel my personal life is being invaded by this technology), techno-complexity (five items, e.g., I do not know enough about this technology to handle my job satisfactorily), techno-insecurity (four items, e.g., I have to constantly update my skills to avoid being replaced), and techno-uncertainty (four items, e.g., There are always new developments in the technologies we use in our organization). Scale items were measured on a five-point scale ranging from 1 (“*strongly disagree*”) to 5 (“*strongly agree*”).

Supervisor support was measured by a scale taken from the Q_u_-Bo test, an instrument standardized for the Italian context [43]. The scale included four items (e.g., My supervisor helps employees achieve their goals and grow professionally) with a response scale ranging from 1 (“*strongly disagree*”) to 6 (“*strongly agree*”). The psychometric properties of both self-report instruments were investigated, and the results are reported in the following Confirmatory Factor Analysis section.

Concerning biological data, hair samples were collected painlessly and non-invasively from the vertex posterior region of the head, and then stored at room temperature in a paper envelope and protected from UV rays until processing. The whole procedure is described in detail elsewhere (see Falco et al.) [44]. Briefly, twenty-five milligrams of hair were weighted, and each hair strand was washed twice using H2O for 3 min and then twice with isopropanol for 3 min, in accordance with Davenport et al. [45]. Cortisol was extracted by incubating each specimen for 16 hours in methanol at 37 °C. Next, the liquid in the vial was evaporated to dryness at 37 °C under an airstream suction hood. The dried residue was then re-suspended in 1.2 ml of ELISA buffer (50 mM phosphate buffer, pH 7.4, 0.4% BSA, 0.5 M NaCl). The concentrations of cortisol were measured using an in-house Enzyme-linked Immunosorbent Assay (ELISA) as described by Falco et al. [44].

#### Data analysis.

Two confirmatory factor analysis (CFA) were first run to assess the psychometric properties of the self-report instruments, including factor structure, construct validity, and reliability. The CFAs were carried out using the maximum likelihood estimation with robust standard errors and a scaled test statistic [46]. Model fit was assessed using the chi-square test together with RMSEA, CFI, and SRMR. A non-significant χ^2^ indicated an acceptable model fit, as did values below .08 for RMSEA and SRMR, and values above .90 for CFI [47].

Next, the relationships hypothesized in the study were tested using moderated multiple regression analysis. Five different models were estimated. In Model 1 (M1), HCC were regressed on techno-overload, SS, and the respective interaction term. The other models were similar, except that the technostress creator was techno-invasion in Model 2 (M2), techno-complexity in Model 3 (M3), techno-insecurity in Model 4 (M4), and techno-uncertainty in Model 5 (M5). The independent variables included in M1-M5 were mean-centered, to facilitate the interpretation of the results. If a significant interaction was found, then a simple slope analysis was performed to determine whether the specific technostress creator was associated with HCC at high (+1*SD*) and low (−1*SD*) levels of SS. Additionally, significant interactions were presented graphically [48]. The models were estimated controlling for sex and age [49], as previous studies have shown an association between HCC and these demographic characteristics [50,51]. Finally, HCC was natural log transformed to better approximate normal distribution [52].

### Results

#### Confirmatory factor analysis.

Concerning the TCs scale, a first CFA model showed a moderate fit to data: χ^2^(160) = 249.96, *p* < .001; RMSEA = .08, CFI = .89, SRMR = .09. An examination of the modification indices has shown that the error covariance between the first two items of techno-overload—which shared a similar wording—should be freely estimated. A revised model was then estimated, and fit indices showed an acceptable fit to data: χ^2^(159) = 230.88, *p* < .001; RMSEA = .07, CFI = .91, SRMR = .08. Similarly, the revised model showed a better fit to data compared to the original model, Δχ^2^(1) = 7.34, *p* < .01. Standardized factor loadings ranged from .49 to .88 (median value = .73), and factor correlations ranged from −.06 (techno-insecurity and techno-uncertainty) to .69 (techno-overload and techno-invasion). Finally, values of composite reliability were .76 for techno-overload, .70 for techno-invasion, .86 for techno-complexity, .87 for techno-uncertainty, and .67 for techno-insecurity, the latter being just below the proposed standard .70 [53]. With respect to SS, the CFA model showed a good fit to data: χ^2^(2) = 1.57, *p* = .46; RMSEA = 0, CFI = 1, SRMR = .03. Standardized factor loadings ranged from .71 to .90 (median value = .76) and composite reliability was .86. Overall, the scales aimed at determining TCs and SS in this study showed adequate psychometric properties in terms of factor structure, construct validity, and reliability.

#### Hypothesis testing.

The descriptive statistics and correlations are presented in the a Supplementary Material (S1 Table in S1 File), while the results of the regression analyses (M1-M5) are shown in Table 1.

**Table 1:** Multiple regression analyses for log hair cortisol concentrations (*N* = 102).

Variable	Model 1 Techno-overload		Model 2 Techno-invasion		Model 3 Techno-complexity		Model 4 Techno-insecurity		Model 5 Techno-uncertainty	
	*B*	*SE*	*B*	*SE*	*B*	*SE*	*B*	*SE*	*B*	*SE*
Sex^a^	−0.190	0.152	−0.184	0.153	−0.168	0.153	−0.197	0.152	−0.244^†^	0.145
Age	0.008	0.006	0.007	0.006	0.006	0.006	0.007	0.006	0.005	0.005
Technostress creator^b^	0.001	0.072	0.018	0.064	0.067	0.073	0.060	0.101	0.012	0.065
Supervisor support	0.003	0.058	0.008	0.058	0.008	0.056	0.012	0.058	0.016	0.055
Technostress creator X Supervisor support	0.039	0.052	0.018	0.049	0.022	0.055	−0.054	0.092	−0.153^**^	0.046
*R* ^2^	.046		.042		.049		.047		.141^*^	
Δ*R*^2^	.006		.001		.002		.003		.099^**^	

*Note.* The independent variables included in the models (excluding dichotomous variables) were mean-centered, to enable easier interpretations of the results. The values of hair cortisol concentration were log-transformed prior to data analysis.

^a^Female = 0, male = 1.

^b^In each model, a different technostress creator was analyzed. These were techno-overload in Model 1, techno-invasion in Model 2, techno-complexity in Model 3, techno-insecurity in Model 4, and techno-uncertainty in Model 5.

† *p* < .10.

* *p* < .05.

** *p* < .01.

In M1-M4 the predictors did not account for a significant proportion of variation in log HCC, with *R*^2^ ranging between .04 and .05. In these models, techno-overload (M1), techno-invasion (M2), techno-complexity (M3), and techno-insecurity (M4) were not associated with log HCC. Similarly, the interaction terms between SS and each techno stressor in M1-M4 were not significant. A different picture emerged for M5, in which the predictors accounted for 14.1% of variation in log HCC, *R*^2^ = .14, *F*(5, 96) = 3.14, *p* = .01. In this model, techno-uncertainty was not associated with log HCC, *b* = 0.01, *SE* = 0.07, *p* = .85. However, the interaction term between techno-uncertainty and SS was significant, *b* = −0.15, *SE* = 0.05, *p* < .01, and accounted for an additional 10% of the variance in log HCC, *F*_change_(1, 96) = 11.06, *p* < .01, *f*^2^ = .12, considered a small to medium effect [48]. Simple slope analysis showed that the association between techno-uncertainty and log HCC was positive and significant when SS was low (*b* = 0.20, *SE* = 0.08, *p* = .02) but negative—albeit marginally significant—when SS was high (*b* = −0.18, *SE* = 0.09, *p* = .05). Specifically, when SS was low, each unit increase in techno-uncertainty was associated with a 22.4% increase in HCC. Conversely, when SS was high, each unit increase in techno-uncertainty was associated with a 16.2% decrease in HCC [52]. The interaction between techno-uncertainty and SS is shown in the Supplementary Material (S2 Fig in S1 File). Overall, H1 was not supported, while H2 was supported only for techno-uncertainty.

### Discussion

Overall, our study provides little support for the association between TCs and HCC, and therefore hypothesis 1 was not supported. Interestingly, a different, more nuanced picture emerged for techno-uncertainty. Although the constant change in work-related technologies appeared to be not associated with HCC at average levels of SS, techno-uncertainty was positively associated with HCC when SS was low, but this association was negative—albeit marginally significant—when SS was high. Therefore, our study suggests that SS has a significant impact on the way remote workers appraise and/or cope with the constant change of new technologies, with implications for the associated physiological costs. Hypothesis 2 was partially supported for techno-uncertainty. Our results in the technostress domain are in line with biological research findings. These findings show that social support may reduce the human cortisol response in stressful situations that are free of technology [54,55].

## Study 2: Psychophysical strain assessed by occupational physician

As noted in the introduction, TCs—conceptualized as job demands—require effort and drain workers’ psychological and physical resources (e.g., attention, energy), possibly leading to psychophysical symptoms over time [8], especially when employees do not have sufficient opportunities for recovery [19,37]. Past research has shown that TCs are related to increased psychophysical symptoms associated with work-related stress [10], including for example exhaustion and fatigue [19,23], both in the general working population but also among remote workers [56].

However, as noted by Fischer and Riedl [20] (see also Nastjuk et al. [19]), most studies on technostress have adopted self-report measures to investigate both TCs and the associated psychological, physiological, and behavioral outcomes for the individuals. Although this type of measurement offers a wide range of relevant applications for research on technostress, empirical studies support the need for a multi-method approach that combines different measurement methods [34], including psychophysical strain as measured by the occupational physician through hetero-evaluations (see, for an example Falco et al. [57]). Accordingly, in this study we combined psychological and psychophysical data—collected through self- and hetero-evaluations—to determine TCs and psychophysical strain, respectively. We expected TCs to be positively associated with psychophysical strain detected by the occupational physician.

Hypothesis 3 (H3): Technostress creators, in terms of techno-overload, techno-invasion, techno-complexity, techno-insecurity, techno-uncertainty, will be positively associated with psychophysical strain determined by the occupational physician.

### Virtual leadership

Leaders play an influential role in determining the destiny of organizations through their decisions and influence on others [58]. Researchers in the field of work and organizational psychology have explored the role of leadership in the JD-R theory to understand its impact on occupational health and performance [32]. By being at a higher level than the dimensions usually considered in the JD-R theory (e.g., job demands/resources), leadership can be conceived as a construct that plays different roles, such as attenuating the association between job demands and strain [32]. Virtual leadership (or e-leadership) [13,14] can be defined as a process of social influence mediated by ICTs with the goal of creating changes in attitudes, feelings, thinking, behavior, and/or performance which can occur at different hierarchical levels in an organization (e.g., individuals, groups, or organizations) [59]. In particular, VL requires leaders to develop specific skills to improve the functioning of the organization in a virtual working environment [12]. An effective virtual team leader is one who is able to use ICTs to build and maintain trust as well as reduce the perceptions of isolation among team members, set clear goals, achieve results by adapting to circumstances and, above all, communicate effectively with digitally collaborating employees [13,14,60]. Specifically, a well-managed communication can be essential for virtual leaders to coordinate tasks, help prevent or reduce conflict while fostering positive relationships, and ultimately increase team effectiveness [60]. The virtual leader can also help to reduce the feelings of exhaustion that employees may feel as a consequence of intensive video conferencing (i.e., zoom fatigue) [61–63], which has been shown to increase depression and burnout tendencies [64], among others. However, a virtual leader may not necessarily be able to reduce TCs directly, but he/she can help employees to deal with them by providing resources (technology-related job resources) [33], thus reducing negative consequences in terms of psychophysical strain [32]. In addition, by fostering trust, social inclusion, and team cohesion [14], a virtual leader can support employees in sustaining their technology-related efforts, which are perceived as worthwhile and directed towards a greater purpose (e.g., the good of the team) [32], containing the psychophysiological costs for the individual and, eventually, psychophysical strain. In line with this theoretical foundation and existing empirical evidence, in this study we hypothesized that VL would buffer the association between TCs and psychophysical strain detected by the occupational physician.

Hypothesis 4 (H4): Virtual leadership will attenuate the relationship between technostress creators, in terms of techno-overload, techno-invasion, techno-complexity, techno-insecurity, and techno-uncertainty, and psychophysical strain detected by the occupational physicians, which is expected to be weaker when virtual leadership is high.

### Materials and methods

#### Procedures and participants.

Participants were employees of a private company in the service sector who worked remotely for all or part of their working time. Specifically, 108 workers agreed to take part in a research project aimed at sustaining workers’ wellbeing during the COVID-19 pandemic. The recruitment period started on 22 June 2022 and ended on 31 July 2022. In the first phase of the study (end of June 2022) participants completed a self-report questionnaire (i.e., psychological data) designed to determine TCs, VL as well as sociodemographic variables (sex, age, occupation, seniority, and parental status). Employees were also given a personal identification code that was necessary to match psychological data (i.e., self-evaluations) with psychophysical data collected by the occupational physician (i.e., hetero-evaluations). Before completing the questionnaire, workers were informed that their participation in the study was voluntary and confidential, and that they were free to withdraw at any time. Participants also provided written informed consent before data collection. Next, in July 2022 employees underwent an occupational health examination carried out by the occupational physician as part of the scheduled occupational health surveillance. Briefly, the physician informed workers the purpose of the investigation was to detect possible psychological and physical symptoms linked to psychophysical strain, which were then assessed by administering an ad-hoc instrument during a structured interview. The study was performed in line with the principles of the Declaration of Helsinki. The project was approved by the Psychological Research Ethics Committee (Area 17), Department/Section of Psychology – University of Padua, Italy (protocol n. 4632).

Overall, three participants (2.8%) had extensive missing data in the self-report questionnaire and were therefore excluded from the study. Therefore, the final sample consisted of 105 participants. Sixty-six participants were male (62.9%) and 39 female (37.1%) with most respondents being under 50 years of age (64.8%). In terms of their occupation, 78 participants were white-collar workers (74.3%) and 24 were middle or top managers (22.9%; three missing values). Finally, most of the participants had children (57.1%; three missing values) and had been with the company for more than 5 years (73.3%; three missing values).

#### Measures.

With respect to psychological data, this study used shortened versions of established self-report questionnaires. As previous research has shown, a longer questionnaires can reduce response rates in various contexts possibly leading to non-response bias [65], and short scales are frequently used [66]. The psychometric properties of the self-report instruments were investigated, and main results are reported in the following Confirmatory Factor Analysis section.

Technostress creators were determined using a shortened version of an Italian adaptation of the questionnaire developed by Ragu-Nathan et al. [17]. The scale included 14 items (see Study 1) that measured techno-overload (two items), techno-invasion (three items), techno-complexity (three items), techno-insecurity (two items), and techno-uncertainty (four items), with a response scale ranging from 1 (“*strongly disagree*”) to 5 (“*strongly agree*”).

Virtual leadership was measured by an adapted version of the Global Virtual Team Leadership Scale [60]. The scale included eight items addressing leadership of supervisors in virtual teams (e.g., The virtual team leader determines the meeting time, taking into account the availability and private lives of the team members). The response scale ranged from 1 (“*strongly disagree*”) to 5 (“*strongly agree*”).

Concerning psychophysical data, the occupational physician determined psychophysical strain using a short version of the Psychophysical Health Inventory [57]. The instrument included 14 items that measured the occurrence of both psychological (e.g., feeling sad or depressed, asthenia) and physical (e.g., headache, eye strain) symptoms linked to work-related stress during the previous six months, with a response scale ranging from 1 (“*never*”) to 6 (“*daily*”).

#### Data analysis.

First, two CFA models were first run to assess the psychometric properties of the self-report instruments (see Study 1 for details). Next, five moderated multiple regression models were estimated to test the relationships hypothesized in the study. In Model 1 (M1), psychophysical strain was regressed on techno-overload, VL, and the respective interaction term. The other models were similar, except that the TC was techno-invasion in Model 2 (M2), techno-complexity in Model 3 (M3), techno-insecurity in Model 4 (M4), and techno-uncertainty in Model 5 (M5). The models were estimated controlling for sex and age, as previous studies have shown an association with psychophysical strain [67–69]. Finally, psychophysical strain was natural log transformed to better approximate normal distribution [52]. For methodological details see Study 1.

### Results

#### Confirmatory factor analysis.

Concerning the shortened version of the TCs scale, a CFA model showed an acceptable fit to data: χ^2^(67) = 105.09, *p* < .01; RMSEA = .07, CFI = .94, SRMR = .07. Standardized factor loadings ranged from .42 to .96 (median value = .80), and factor correlations ranged from −.02 (techno-invasion and techno-uncertainty) to .70 (techno-complexity and techno-insecurity). Finally, values of composite reliability were .92 for techno-overload, .81 for techno-invasion, .83 for techno-complexity, .83 for techno-insecurity, and .82 for techno-uncertainty. For the VL scale, a first CFA model showed a mediocre fit to data: χ^2^(20) = 65.86, *p* < .001; RMSEA = .15, CFI = .78, SRMR = .10. An examination of the modification indices has shown that the error covariance between two couples of items—which shared a similar wording—should be freely estimated. A revised model was then estimated, and fit indices showed an acceptable fit to data: χ^2^(18) = 28.97, *p* = .05; RMSEA = .08, CFI = .95, SRMR = .06. Similarly, the revised model showed a better fit to data compared to the original model, Δχ^2^(2) = 28.40, *p* < .001. Standardized factor loadings ranged from .48 to .87 (median value = .59), and the value of composite reliability was .80.

#### Hypothesis testing.

The descriptive statistics and correlations are presented in the Supplementary Material (S3 Table in S1 File), while the results of the regression analyses (M1-M5) are shown in Table 2.

**Table 2:** Multiple regression analyses for psychophysical strain (*N* = 105).

Variable	Model 1 Techno-overload		Model 2 Techno-invasion		Model 3 Techno-complexity		Model 4 Techno-insecurity		Model 5 Techno-uncertainty	
	*B*	*SE*	*B*	*SE*	*B*	*SE*	*B*	*SE*	*B*	*SE*
Sex ^a^	−0.085^†^	0.049	−0.082^†^	0.048	−0.085^†^	0.051	−0.085^†^	0.048	−0.108^*^	0.051
Age ^b^	0.024	0.051	0.051	0.049	0.040	0.053	0.035	0.049	0.035	0.053
Technostress creator ^c^	0.037	0.027	0.099^**^	0.029	−0.004	0.030	0.014	0.037	−0.036	0.034
Virtual leadership	−0.076^†^	0.043	−0.047	0.044	−0.088^*^	0.044	−0.053	0.045	−0.126^**^	0.041
Technostress creator X Virtual leadership	−0.102^*^	0.043	−0.082^*^	0.038	−0.085^*^	0.033	−0.155^***^	0.045	−0.036	0.057
*R* ^2^	.180^**^		.235^***^		.176^**^		.234^***^		.140^**^	
Δ*R*^2^	.046^*^		.035^*^		.054^*^		.091^***^		.004	

*Note.* The independent variables included in the models (excluding dichotomous variables) were mean-centered, to enable easier interpretations of the results. The values of psychophysical strain were log-transformed prior to data analysis.

^a^Female = 0, male = 1.

^b^≤ 50 years old = 0, > 50 years old = 1.

^c^In each model, a different technostress creator was analyzed. These were techno-overload in Model 1, techno-invasion in Model 2, techno-complexity in Model 3, techno-insecurity in Model 4, and techno-uncertainty in Model 5.

† *p* < .10.

* *p* < .05.

** *p* < .01.

*** *p* < .001.

In all the models tested the predictors accounted for a significant proportion of variation in log psychophysical strain, with *R*^2^ ranging between .14 (techno-uncertainty) and .24 (techno-invasion). Among TCs, only techno-invasion in M2 was positively associated with log psychophysical strain at average levels of VL and controlling for the effect of sex and age, *b* = 0.10, *SE* = 0.03, *p* < .01. Specifically, each unit increase in techno-invasion was associated with a 10.4% increase in psychophysical strain at average levels of VL. Therefore, H3 was only supported for techno-invasion.

The interaction between techno-overload and VL was significant in M1, *b* = −0.10, *SE* = 0.04, *p* = .02, and accounted for an additional 4.6% of the variance in log psychophysical strain, F_change_(1, 99) = 5.54, *p* = .02, *f*^2^ = .06, a small to medium effect. Simple slope analysis showed that the association between techno-overload and log psychophysical strain was positive and significant when VL was low (*b* = 0.10, *SE* = 0.04, *p* = .01) but not significant when VL was high (*b* = −0.02, *SE* = 0.04, *p* = .51). Specifically, when VL was low, each unit increase in techno-overload was associated with a 10.5% increase in psychophysical strain. The interaction between techno-overload and VL is shown in the Supplementary Material (S4 Fig in S1 File).

Techno-invasion and techno-insecurity showed a similar pattern of results. The interaction term between techno-invasion and VL was negative and significant in M2, *b* = −0.08, *SE* = 0.04, *p* = .04, and accounted for an additional 3.5% of the variance in log psychophysical strain, F_change_(1, 99) = 4.55, *p* = .04, *f*^2^ = .05, a small to medium effect. Simple slope analysis showed that the association between techno-invasion and log psychophysical strain was positive and significant when VL was low (*b* = 0.15, *SE* = 0.04, *p* < .001) but not significant when VL was high (*b* = 0.05, *SE* = 0.04, *p* = .17). Each unit increase in techno-invasion was associated with a 16% increase in psychophysical strain when VL was low. The interaction between techno-invasion and VL is shown in the Supplementary Material (S5 Fig in S1 File). The interaction term between techno-insecurity and VL was negative and significant in M4, *b* = −0.16, *SE* = 0.05, *p* < .001, and accounted for an additional 9.1% of the variance in log psychophysical strain, F_change_(1, 99) = 11.79, *p* < .001, *f*^2^ = .12, a small to medium effect. Simple slope analysis showed that the association between techno-insecurity and log psychophysical strain was positive and significant when VL was low (*b* = 0.11, *SE* = 0.04, *p* < .01) but not significant when VL was high (*b* = −0.08, *SE* = 0.05, *p* = .14). When VL was low each unit increase in techno-insecurity was associated with a 11.3% increase in psychophysical strain. The interaction between techno-insecurity and VL is shown in the Supplementary Material (S6 Fig in S1 File).

Finally, the interaction term between techno-complexity and VL was negative and significant in M3, *b* = −0.09, *SE* = 0.03, *p* = .01, and accounted for an additional 5.4% of the variance in log psychophysical strain, F_change_(1, 99) = 6.54, *p* = .01, *f*^2^ = .07, a small to medium effect. Simple slope analysis showed that the association between techno-complexity and log psychophysical strain was not significant either when VL was low (*b* = 0.05, *SE* = 0.03, *p* = .16) or when VL was high (*b* = −0.06, *SE* = 0.04, *p* = .15). A closer look at the regions of significance showed that the association between techno-complexity and log psychophysical strain would be positive and significant for VL values lower than 3.01, a rather extreme value (i.e., roughly 1.65 SD below the mean of VL). Accordingly, H4 was partially supported.

### Discussion

Overall, a complex pattern of results emerged in Study 2. Only techno-invasion was positively related to psychophysical strain determined by the occupational physician at average levels of VL, with this association being medium-sized (semi-partial correlation = .30). In addition, VL appeared to play a relevant role in the associations between some, but not all, TCs and psychophysical strain. Specifically, VL attenuated the association between techno-overload, techno-invasion, and techno-insecurity and psychophysical strain, with this association being positive and significant when VL was low, but not significant when VL was high. This implies that SWs benefit from higher levels of VL because they are more likely to be able to deal with techno-overload/-invasion/-insecurity effectively, thus reducing the associated psychophysical costs and—ultimately—psychophysical strain.

## General discussion

The objective of this paper was to contribute to a better understanding of the consequences of technostress in SWs. In contrast to previous research that often relied on self-report measurement and hence subjective evaluations (see a recent meta-analysis by Nastjuk et al. [19]), our studies revealed a nuanced pattern of results. Study 1 found limited evidence of an association between established technology-related risk factors and hair cortisol as a biomarker of stress [9]. However, techno-uncertainty showed a different association with HCC (i.e., positive *vs*. negative) depending on the level of supervisor support (i.e., low *vs*. high).

When interpreting these findings, it is important to keep in mind that in stressful situations, cortisol release and HPA axis activation in general are particularly driven by three factors: (i) important personal goals are threatened, (ii) the situation is uncontrollable, and/or (iii) task performance may be judged negatively by others [70]. Specifically, while conditions that threaten the social self can elicit negative self-evaluations and diminish one’s standing in the eyes of others [71], uncontrollability may impede progress towards the attainment of relevant (work-related) goals [70]. However, as conceptualized and measured in this study, the TCs likely did not directly include a strong social-evaluative component [72], which may be particularly true for employees working remotely. Furthermore, although some TCs may be perceived as uncontrollable to a certain extent (e.g., techno-complexity), it should be noted that SWs make extensive and continuous use of ICTs to perform work-related tasks and hence they are typically experienced users. Consequently, they can anticipate the occurrence of certain technology-related stress situations when working remotely and be prepared to deal with them effectively [73]. It follows that SWs may view TCs to some extent as an unavoidable, legitimate, predictable or even self-chosen part of the job that needs to be managed effectively [72]. Technostress creators may represent the “dark side” of ICTs use, which, as the “bright side”, may be functional to achieve relevant goals for the individual both at work (e.g., job performance) [74] and in the private life (e.g., work-life balance through remote working) [75]. Thus, this relative lack of a social-evaluative component and uncontrollability of our operationalization of TCs may provide a tentative explanation for the lack of association between TCs (with the exception of techno-uncertainty, as discussed below) and HCC in the current study. However, future research is needed to corroborate and extend our findings. Ideally, such future studies would be based on research designs that deliberately manipulate the three factors target threat, uncontrollability, and social-evaluative [70] as independent variables in order to then determine the effects of such manipulation on dependent variables such as physiological stress (e.g., cortisol, heart rate variability), users’ subjective stress perceptions, and hetero-evaluations in relation to stress symptoms. Interestingly, techno-uncertainty may be regarded as an exception, as it was associated with HCC in a different direction depending on the level of support received from one’s supervisor. Specifically, techno-uncertainty was positively related to HCC under low support conditions, but negatively related to HCC under high support conditions. Common attributes of techno-uncertainty include feeling loss of control due to constant changes in the software/hardware/networks or IS policies, which are mostly driven by the organization [76]. Nevertheless, it is possible that such changes are not always perceived by employees as negative, stress-inducing conditions [19]. Rather, in certain circumstances change can create a stimulating context in which users are able to appreciate the value of new technology in facilitating task fulfillment [77]. Notably, previous research on work-related stress suggests that job demands may contain both challenging (i.e., promoting the accomplishment of work tasks and personal development) and hindering (i.e., thwarting the accomplishment of job tasks or the opportunity to achieve work goals) components, which have different consequences for individual well-being [78,79]. In addition, job resources such as social support may alter how job demands are appraised as challenging *vs*. hindering [79]. Specifically, job demands may be more strongly appraised as challenges under conditions of high social support, but more strongly appraised as hindrances when social support is low, with different consequences on individual well-being [80]. In summary, when supported by their supervisor, SWs can perceive techno-uncertainty as a challenge technostressor, where changes in technology are appraised as opportunity to improve task accomplishment, leading to positive outcomes and lower HCC (i.e., techno-eustress) [73]. In contrast, when supervisor support is limited, SWs may perceive technology change as hindrance technostressor—a barrier or obstacle to task accomplishment—related to negative outcomes and increased HCC (i.e., techno-distress) [73]. This explanation is consistent with the idea that uncontrollability is not necessarily associated with cortisol responses, but only when uncontrollability poses a substantial threat to a central goal for the individual [70].

To the best of our knowledge, the only one study that investigated the association between TCs and hair cortisol provided preliminary indications for alterations of the HPA axis activity—in terms of reduced HCC—among hospital employees [72]. In addition, we emphasize that the TCs in this study were conceptualized and measured differently than in our study. Specifically, Kaltenegger et al. [72] examined work interruptions, multitasking, and information overload. Therefore, these differences in conceptualization and measurement may explain the differences in findings. In addition, it is possible that the different user groups (this article, Study 1: employees from different organizations *vs*. hospital employees in Kaltenegger et al. [72]) show systematic differences in their personality and work contexts, which may also explain differences in the results. Moreover, we highlight that a recent cross-sectional study reported a positive association between technostress and serum cortisol among university medical staff members and students [81]. However, it should be noted that these studies showed noticeable differences in terms of research design and conceptualizations of TCs, which make it difficult again to draw firm conclusions. Therefore, further research is warranted.

Study 2 showed that techno-overload, techno-invasion, and techno-insecurity were positively associated with psychophysical strain determined by the occupational physician when VL was low, with techno-invasion also being positively associated with strain at average levels of VL. These findings prompt two considerations.

First, although the results of Study 1 and Study 2 may appear to contradict each other, it should be noted that stress has an impact on a number of biological systems, which interact with each other in order to promote adaptation in the face of threat situations [82]. These include the HPA axis but also the autonomic nervous system and the immune systems, and previous research has demonstrated that stressful situations may result in dysregulations in any of these systems, which can in turn result in stress-related health outcomes [35]. Research published between the 1970s (when the first PCs were deployed in organizations) and the 2010s on these interacting biological systems is comprehensively discussed in a review of the biology of technostress [25]. Importantly, recent research has also addressed this topic. For example, in a laboratory setting Becker et al. [83] observed that salivary alpha-amylase concentrations—as a marker for the sympathetic nervous system reactivity—significantly changed in individuals exposed to work interruptions and multitasking conditions, while no significant changes were observed for cortisol, Immunoglobulin-A and C-reactive protein as markers of the HPA axis and the immune system, respectively. Taken together, our findings indicate that TCs are associated with the stress response, but also underscore the need for further research into the biological underpinnings of stress and technostress. Understanding the circumstances under which TCs elicit specific psychophysiological responses would contribute to a deeper understanding of how repeated or chronic (techno)stressors “get under the skin” [72] and lead to health impairment.

Second, our study showed that VL can attenuate the association between TCs and the strain response. This again highlights the pivotal role of the positive relationship with one’s supervisor in reducing the level of technostress experienced by users. As a tentative explanation, VL may affect how SWs appraise technology-related stressful situations, which are appraised as hindering (*vs*. challenging) technostressors when VL is low (*vs*. high) [73]. In addition, a virtual leader can help employees to deal with TCs by providing adequate technology-related job resources [33]. Examples include shared and mutually agreed silent times in workplaces and designated days of the week for teleworking or meetings. In both cases, VL can help to reduce the negative effects of TCs in terms of psychophysical strain. However, future research is needed to arrive at more definitive conclusions.

Some limitations should be considered when interpreting the results of the present study. First, both studies were cross-sectional in nature, so the temporal order of the investigated variables (a precondition for causal inferences) could not be assessed directly. This is particularly relevant in relation to HCC, as timing plays a central role in causal inferences with respect to the association between stressful situations and HPA axis activity [71]. Second, previous studies have indicated that probably a certain stress (i.e., workload) threshold needs to be reached to detect a relationship between self-reported stressful situations at work and changes in hair cortisol [84]. However, in our study the levels of TCs appeared to be relatively low, compared to past research [17]. Third and finally, our study was conducted in March/April 2022, two years after the first full national lockdown due to the COVID-19 pandemic. While many employees were forced and unprepared to work from home during the lockdown, SWs in our sample likely had prior experience with remote work and had access to appropriate home office equipment [66]. This again may have mitigated negative consequences of technostress.

## Conclusion

The increasing use of ICTs in both private and professional contexts suggests that more and more people are likely to experience technology-related outcomes over time, both positive (e.g., increased efficiency and innovation) and negative (e.g., technostress) [15]. Albeit technology can have both a “bright” and a “dark” side, technology-related risk factors—if not properly understood and managed—can lead to impaired health and reduced job satisfaction in the workforce [19]. At the same time, they can dampen the potential advantages that organizations can derive from their ICTs investments and undermine organizational effectiveness [74]. Therefore, addressing technostress would be a key asset for organizations to improve the well-being and job performance of employees, especially those working remotely [81]. By showing an association between TCs and the strain response mostly when support from one’s supervisor and VL were low, our study sheds light on mechanisms potentially linking technostress and disease in a population of workers who are particularly at risk. Although more research is certainly needed, our study contributes to the identification of a panel of possible biomarkers of technostress, similarly to the composite AL index [85]. Such a tool would be extremely useful for organizations and practitioners in the early detection of technostress and the prevention of more serious health consequences. In terms of practical implications, this study suggests that supervisors in virtual settings should be encouraged to engage in both task-oriented and relational-oriented leadership behaviors. In addition, practitioners should emphasize skills development for virtual team leaders (e.g., virtual communication training), with the aim to help team members to effectively manage technology-related stressful situations when working remotely.

## Supporting information

S1 File Supporting information. (ZIP)
