# Supplementary material for: Supervisor support and virtual leadership moderate the association between technostress creators and strain in remote work: Evidence based on hair cortisol and occupational physician’s hetero-evaluations
Source: PLoS One. 2025 Jun 13;20(6):e0323385. doi: 10.1371/journal.pone.0323385 (PMC12165403; doi:10.1371/journal.pone.0323385)
Supplement: S1 File — (ZIP) [file pone.0323385.s001.zip › S1 Table.pdf]

Supervisor support and virtual leadership moderate the association between technostress creators and strain in remote work: Evidence based on hair cortisol and occupational physician's hetero-evaluations

Supplementary material

Damiano Girardi[1*], Sebastiano Rapisarda[1¶], Elvira Arcucci[1¶], Laura Dal Corso[1], René Riedl[2,3], Isabella Pividori[4], Alessandra Falco[1]

[1] Department of Philosophy, Sociology, Education and Applied Psychology, University of Padua, Padua, Italy

[2] Digital Business Institute, University of Applied Sciences Upper Austria, Campus Steyr, Steyr, Austria

[3] Institute of Business Informatics – Information Engineering, University of Linz, Linz, Austria

[4] Department of Agricultural, Environmental and Animal Sciences, University of Udine, Udine, Italy

* Corresponding author

E-mail: damiano.girardi@unipd.it

ORCID: https://orcid.org/0000-0002-1326-9215

[¶] These authors contributed equally to this work.

Table 1. Means, Standard Deviations and Correlations for Study Variables (*N* = 102)

Variable	*M*	*SD*	1	2	3	4	5	6	7	8	9
1. Hair cortisol (pg/mg) [a]	11.76	9.93	—								
2. Techno-overload	2.28	0.95	.03	—							
3. Techno-invasion	2.32	1.07	.05	.59**	—						
4. Techno-complexity	2.10	0.94	.14	.44**	.34**	—					
5. Techno-insecurity	1.63	0.67	.03	.33**	.18	.35**	—				
6. Techno-uncertainty	2.52	1.01	.05	.07	.06	.11	−.06	—			
7. Supervisor support	4.01	1.24	−.05	−.25*	−.25*	−.06	−.18	.19	—		
8. Sex [b]	0.29	0.46	−.15	−.11	−.16	−.19	.06	.12	.28**	—	
9. Age	39.34	12.18	.15	.19	.03	.20*	−.10	.18	−.21*	−.20*	—

Note. *M* and *SD* are used to represent mean and standard deviation, respectively.

[a] Hair cortisol concentration. Values were log-transformed prior to data analysis, including the correlations shown above. [b] Female = 0, male = 1.

$^{*}p < .05$. $^{**}p < .01$.
