# Supplementary material for: Supervisor support and virtual leadership moderate the association between technostress creators and strain in remote work: Evidence based on hair cortisol and occupational physician’s hetero-evaluations
Source: PLoS One. 2025 Jun 13;20(6):e0323385. doi: 10.1371/journal.pone.0323385 (PMC12165403; doi:10.1371/journal.pone.0323385)
Supplement: S1 File — (ZIP) [file pone.0323385.s001.zip › S3 Table.pdf]

Supervisor support and virtual leadership moderate the association between technostress creators and strain in remote work: Evidence based on hair cortisol and occupational physician's hetero-evaluations

Supplementary material

Damiano Girardi[1*], Sebastiano Rapisarda[1¶], Elvira Arcucci[1¶], Laura Dal Corso[1], René Riedl[2,3], Isabella Pividori[4], Alessandra Falco[1]

[1] Department of Philosophy, Sociology, Education and Applied Psychology, University of Padua, Padua, Italy

[2] Digital Business Institute, University of Applied Sciences Upper Austria, Campus Steyr, Steyr, Austria

[3] Institute of Business Informatics – Information Engineering, University of Linz, Linz, Austria

[4] Department of Agricultural, Environmental and Animal Sciences, University of Udine, Udine, Italy

* Corresponding author

E-mail: damiano.girardi@unipd.it

ORCID: https://orcid.org/0000-0002-1326-9215

[¶] These authors contributed equally to this work.

Table 2. Means, Standard Deviations, and Correlations for Study Variables (*N* = 105)

Variable	*M*	*SD*	1	2	3	4	5	6	7	8	9
1. Psychophysical strain [a]	1.38	0.41	—								
2. Techno-overload	2.08	0.93	.22*	—							
3. Techno-invasion	1.63	0.81	.36**	.48**	—						
4. Techno-complexity	1.97	0.94	.19	.46**	.34**	—					
5. Techno-insecurity	1.61	0.77	.29**	.50**	.27**	.58**	—				
6. Techno-uncertainty	2.84	0.78	−.06	.34**	.03	.21*	.23*	—			
7. Virtual leadership	4.01	0.60	−.28**	−.31**	−.26**	−.36**	−.49**	−.11	—		
8. Sex [b]	0.63	0.49	−.19	−.09	−.10	−.25*	−.07	−.16	−.02	—	
9. Age [c]	0.35	0.48	.13	.14	.04	.34**	.22*	.12	−.17	−.09	—

Note. M and *SD* are used to represent mean and standard deviation, respectively. [a] Values were log-transformed prior to data analysis, including the correlations shown above. [b] Female = 0, male = 1. [c] ≤ 50 years old = 0, > 50 years old = 1.

* $p < .05$. ** $p < .01$.
