# Supplementary material for: Supervisor support and virtual leadership moderate the association between technostress creators and strain in remote work: Evidence based on hair cortisol and occupational physician’s hetero-evaluations
Source: PLoS One. 2025 Jun 13;20(6):e0323385. doi: 10.1371/journal.pone.0323385 (PMC12165403; doi:10.1371/journal.pone.0323385)

Supervisor support and virtual leadership moderate the association between technostress creators and strain in remote work: Evidence based on hair cortisol and occupational physician's hetero-evaluations

Supplementary material

Damiano Girardi[1*], Sebastiano Rapisarda[1¶], Elvira Arcucci[1¶], Laura Dal Corso[1], René Riedl[2,3], Isabella Pividori[4], Alessandra Falco[1]

[1] Department of Philosophy, Sociology, Education and Applied Psychology, University of Padua, Padua, Italy

[2] Digital Business Institute, University of Applied Sciences Upper Austria, Campus Steyr, Steyr, Austria

[3] Institute of Business Informatics – Information Engineering, University of Linz, Linz, Austria

[4] Department of Agricultural, Environmental and Animal Sciences, University of Udine, Udine, Italy

* Corresponding author

E-mail: damiano.girardi@unipd.it

ORCID: https://orcid.org/0000-0002-1326-9215

[¶] These authors contributed equally to this work.

Fig. 2. The Moderating Role of Virtual Leadership in the Relationship Between Techno-Overload and Log Psychophysical Strain.

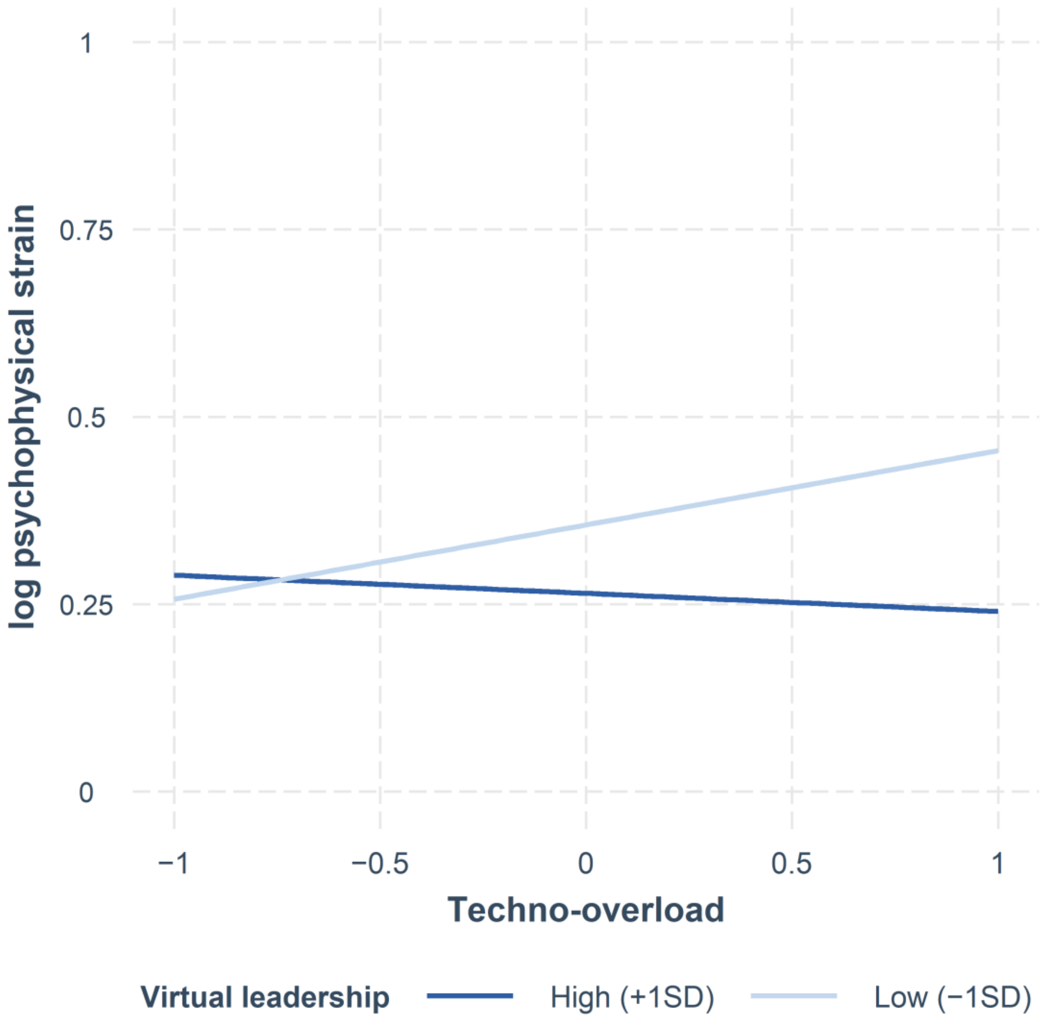

Supplement: S1 File — (ZIP) [file pone.0323385.s001.zip › S4 Fig.pdf]
